# Identification of sesquiterpene synthases from the Basidiomycota *Coniophora puteana* for the efficient and highly selective β-copaene and cubebol production in *E. coli*

**DOI:** 10.1186/s12934-018-1010-z

**Published:** 2018-10-22

**Authors:** Wolfgang Mischko, Max Hirte, Monika Fuchs, Norbert Mehlmer, Thomas B. Brück

**Affiliations:** Werner Siemens-Chair of Synthetic Biotechnology, Department of Chemistry, Technical University of Munich, 85748 Garching, Germany

**Keywords:** Copaene, Cubebol, Sesquiterpene, Basidiomycota, *Coniophora puteana*, Heterologous expression, Fermentation, Phylogenetic analysis

## Abstract

**Background:**

Terpenes are an important and extremely versatile class of secondary metabolites that are commercially used in the pharmaceutical, food and cosmetics sectors. Genome mining of different fungal collections has revealed the genetic basis for a steadily increasing number of putative terpene synthases without any detailed knowledge about their biochemical properties. The analysis and research of this rich genetic source provides a precious basis for the advancing biotechnological production of an almost endless number of valuable natural metabolites.

**Results:**

Three annotated terpene synthases from the little investigated Basidiomycota *Coniophora puteana* were studied in this work. For biochemical characterization, the heterologous expression in *E. coli* was conducted leading to the identification of two sesquiterpene synthases capable of the highly selective generation of β-copaene and cubebol. These compounds are commercially used as food and flavor additives. The new enzymes show the highest reported product selectivity for their main compounds and therefore represent the first exclusive synthases for β-copaene (62% product selectivity) and cubebol (75% product selectivity) generation. In combination with an optimized heterologous microbial production system, we obtained product titers of 215 mg/L β-copaene and 497 mg/L cubebol.

**Conclusion:**

The reported product selectivity and our generated terpene titers exceed all published biotechnological data regarding the production of β-copaene and cubebol. This represents a promising and economic alternative to extraction from natural plant sources and the associated complex product purification.

**Electronic supplementary material:**

The online version of this article (10.1186/s12934-018-1010-z) contains supplementary material, which is available to authorized users.

## Background

Filamentous fungi are experts at producing highly complex natural compounds of commercial interest [1]. Fungal-derived polyketides have been the main focus of recent research activities, whereas the identification of terpenoids and their biosynthesis in fungi have received little attention although these compounds represent the most structurally diverse group of natural products [2]. All terpenoids are based on the same basic C_5_ isoprene building blocks, dimethylallyl pyrophosphate (DMAPP) and isopentenyl pyrophosphate (IPP), which are consecutively fused by head to tail condensation. Depending on their carbon chain length, these linear phosphorylated alkenes are universal precursors of mono(C_10_)-, sesqui(C_15_)-, di(C_20_)-, sester(C_25_)- or tri(C_30_)-terpenes [3]. The structural diversity within the class of terpenoids results from the complex cyclization of the linear precursors into chemically complex molecules, a reaction catalyzed by the family of terpene synthase (TPS) enzymes. More specifically, sesquiterpene synthases (STPSs) transform the linear C_15_ precursor farnesyl pyrophosphate (FPP) into a variety of different scaffolds, which form the structural core of functionalized, bioactive sesquiterpenoids (STPs) [2]. Many STPs are lead structures in pharmaceutical applications, encompassing anti-cancer [4, 5], anti-inflammatory [6, 7] and antibiotic [8] therapies. STPs also have existing applications in the food and cosmetics industries, where they are used as flavor and fragrance ingredients [9, 10].

As the extraction of these latter compounds from natural sources is often cost-intensive and not suitable to meet market demands [11], effective biotechnological production routes are the focus of development efforts [12]. Due to the rapid progress of modern sequencing techniques, in silico genome mining based on conserved amino acid motifs can be applied to identify putative TPSs [13]. Whole genome projects of different mushroom-forming fungi (Basidiomycota) species represent a largely unexplored source of investigation and extraction of rarely characterized STPSs [14, 15]. In this context, numerous putative TPSs have already been annotated but their catalytic capacities remain to be established [14]. To exploit the biotechnological potential of fungal biosynthetic pathways, subsequent functional expression and characterization of these enzymes is required. This represents the first step in providing a sustainable supply of high-value natural products using microorganisms as cell factories [16].

Based on the available genome data, we were able to select potential TPSs from the Basidiomycota *Coniophora puteana*, which is classified as a common wood rotting fungus [17]. At present, only the enzyme systems involved in *C. puteana*-dependent wood depolymerization have been characterized in detail [18, 19]. However, there are no reports of any other enzymes involved in secondary metabolite production. Therefore, this study focuses on the identification and characterization of three putative *C. puteana*-derived TPSs. We present the functional reconstitution of these enzymes in an *E. coli* whole-cell production system. With respect to designing an effective STP production system, the supply of the FPP precursor needed to be ensured by an adapted co-expression of bottleneck enzymes (DXS, Idi) from the native non-mevalonate pathway (MEP) (Fig. 2a). This optimization measure ensured a directed carbon flux towards STP production. Two of the three identified TPSs from *C. puteana* (Copu1-3) could be expressed functionally in *E. coli*, resulting in a range of sesquiterpene products. The main product of the Copu2 fermentations was the tricyclic β-copaene. By contrast, Copu3 fermentations provided cubebol as the main product, which is approved as a dietary supplement and flavoring agent [9, 20] due to its pronounced cooling effect [21]. Utilizing the new terpene synthases from *C. puteana* within an optimized production construct provided 215 mg/L β-copaene and 497 mg/L cubebol. The cubebol production titers reported in this study exceeded all other described production systems by a factor of 50.

## Results

### Identification and characterization of putative terpene synthase genes in C*. puteana*

A Basic Local Alignment Search Tool (BLAST) analysis of fungal genomes with conserved terpene synthases sequences resulted in the identification of a large number of putative terpene synthases (TPSs). However, for the majority of TPS candidates, a biochemical and functional characterization remains to be established. In order to gain insight into their catalytic function, three putative TPSs (Copu1: XP_007772164.1; Copu2: XP_007771895.1; Copu3: XP_007765978.1) from *C. puteana* were selected for cloning and functional characterization. The specific selection was made on the basis of characteristic conserved sequence motifs. Moreover, Copu1-3 showed closely related amino acid (AA) sequences (55–62% similarity). A comparison of the AA sequence of Copu1 and Copu2 with the public database showed < 50% similarity to other listed enzymes, covering all biological realms. By contrast, Copu3 showed 65% similarity to putative TPS sequences, which were not functionally characterized. The AA sequences of all three enzymes contained typical sequence motifs common to the TPS family, such as the highly conserved (N/D)D(L/I/V)x(S/T)xxxE (NSE) triad and the aspartate-rich D(D/E)xxD motif, coordinating a trinuclear Mg^2+^ cluster, which is catalytically essential for the initial hydrolysis of the FPP-derived pyrophosphate group [22] (see Additional file 1). A highly conserved arginine residue, indicated as the pyrophosphate sensor, is located 46 positions upstream of the NSE triad. Additionally, the catalytically important RY-dimer, which is involved in the formation of hydrogen bonds to the substrate-derived pyrophosphate, is found 80 AA downstream of the NSE triad and close to the C-terminus [23–25].

### Heterologous expression of *C. puteana* TPS genes resulted in the generation of diverse sesquiterpenes in *E. coli*

**Fig. 1:**
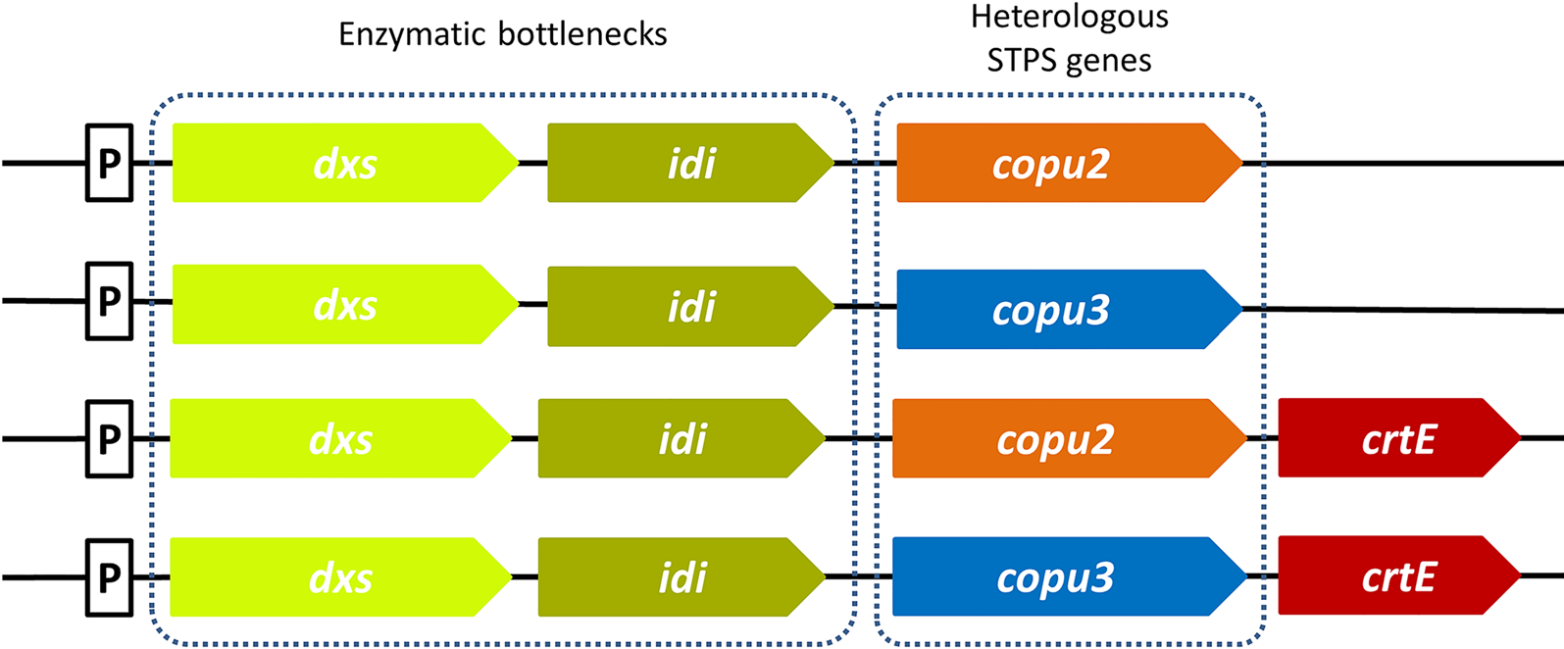
Design of the synthetic operons for an increased precursor supply and efficient STP production. Enzymatic bottlenecks within the upstream non-mevalonate (MEP) pathway (dxs and idi) as well as the heterologous STPS genes (Copu2 and Copu3) are combined in a single synthetic operon. The integration of an additional geranylgeranyl diphosphate synthase (crtE) allowed to study a potential function as diterpene synthases. A constitutive promoter (P) regulates the pathways

**Fig. 2:**
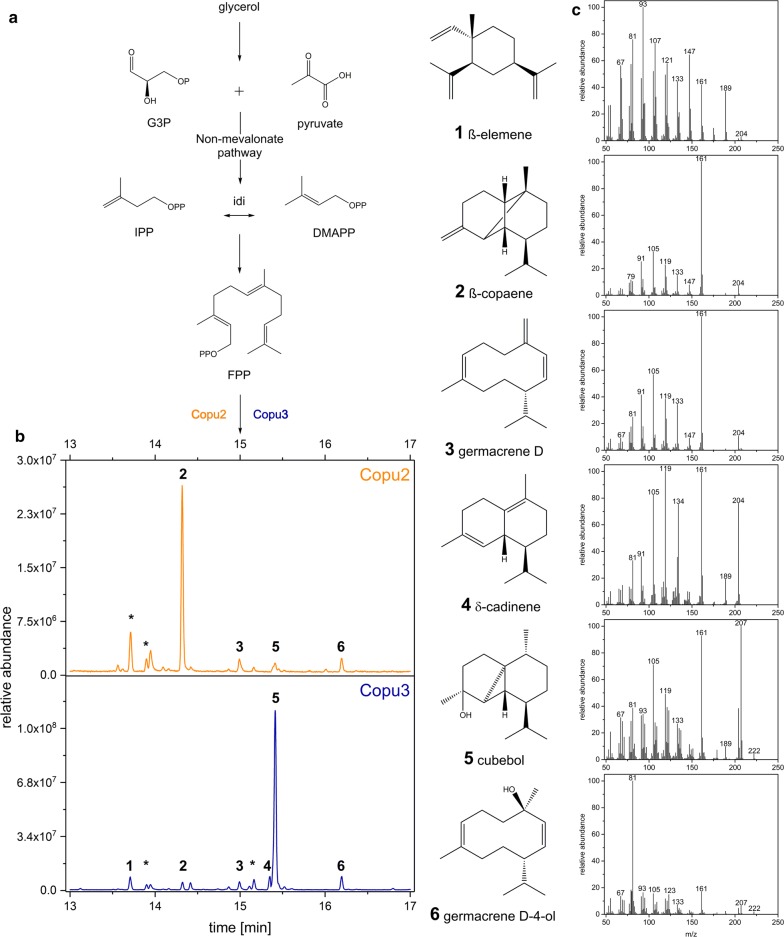
The biosynthetic generation of β-copaene and cubebol. STP production relies on the precursor supply of the non-mevalonate (MEP) pathway (**a**). The respective GC–MS chromatograms of *E. coli* cultures co-expressing either Copu2 or Copu3 and the corresponding MEP bottleneck enzymes reveal new product peaks and distinct product profiles due to the specific cyclization reaction (**b**). The respective detailed MS spectra allow for a putative compound assignment (**c**). The asterisks refers to terpenes without any MS spectra match in the NIST database

To study the product profile of Copu1-3, their predicted coding sequences were codon-optimized and synthesized for transfer into *E. coli* expression vectors. For the heterologous expression, an adapted production system based on a single operon with a constitutive promoter was constructed. Reported bottleneck enzymes from *E. coli*’s native non-mevalonate pathway (MEP) were selected for co-expression (DXS; WP_099145004.1 and idi; AAC32208.1) to increase the precursor supply and enhance the general isoprenoid production (Fig. 1). The resulting plasmids were transformed into *E. coli* HMS174 (DE3) for recombinant gene expression and subsequent analysis of new, potential terpene compounds. In the first series of experiments, a geranylgeranyl diphosphate synthase (*Pantoea ananatis*; crtE; ADD79325.1) was additionally integrated into the operon to study a potential function as diterpene synthases. *E. coli* cultures co-expressing Copu2 or Copu3 with the MEP bottleneck enzymes for 48 h produced a mixture of exclusively 12 and 18 terpene products, respectively. A cultivation temperature of 30 °C and reduced shaking (90 rpm) ensured an adequate bacterial growth rate and the requirements for the production of potentially volatile compounds. Gas chromatography–mass spectrometry (GC–MS) analysis of the liquid extract revealed new product peaks in both cultures with typical mass fragmentation patterns at 161, 207, 222 m/z and 105, 161, 204 m/z (Fig. 2), illustrating the mass patterns for cyclic C_15_ hydrocarbons with and without a single hydroxyl group. Copu3 appeared to be quite selective for the generation of one major STP alcohol (RT: 15.4 min; parent ion at 222 m/z). In contrast, Copu2 appeared to convert FPP into a smaller and slightly different set of cyclization products. The major product of Copu2 fermentations (RT: 14.3 min) was identified as an unhydroxylated STP compound (parent ion at 204 m/z). By contrast, no terpenoid products were detected in *E. coli* cultures expressing Copu1. Neither the co-expression with a GGPP synthase to enable a possible formation of diterpenes nor the evaluation of different fermentation temperatures to avoid eventual evaporation of volatile compounds (e.g., monoterpenes) showed any product formation. Therefore, Copu1 was classified as a non-functional TPS sequence.

### Terpene purification and structure elucidation

A putative identification of the newly generated STPs was performed by comparing their detailed mass spectra to the National Institute of Standards and Technology (NIST) Database. The comparison of the GC–MS metabolite profiles of *E. coli* cultures expressing Copu2 revealed the putative tricyclic STP β-copaene (RT: 14.3 min; parent ion at 204 m/z; major daughter ions at 105, 161 m/z) as the major product. β-Copaene accounted for about 62% of the total STPs detected by GC-FID. *E. coli* strains expressing Copu3 accumulated the putative tricyclic STP alcohol cubebol (RT: 15.4 min; parent ion at 222 m/z; major daughter ions at 105, 161, 207 m/z) as the major product, representing 75% of the total terpene fraction (Fig. 2). Other minor compounds from both cultures encompassed δ-cadinene, β-elemene, germacrene D and germacrene D-4-ol, whose GC–MS spectra were consistent with NIST database references. Notably, several minor STPs did not match any NIST reference spectra and could therefore not be assigned. In addition to GC–MS analyses, the main products of Copu2 and Copu3 were analyzed by NMR spectroscopy. Organic extraction allowed HPLC-based β-copaene and cubebol purification from 1-L fermentations. The HPLC separation of the Copu2 fermentation products allowed for the extraction of the main product but could not resolve several other STPs, which most likely represent a mixture of different product isoforms. However, another minor product was isolated from the Copu3 fermentation broth. A comparison of NMR spectra to reported references confirmed the presence of β-copaene [26, 27], cubebol [28] and δ-cadinene [29] as products of Copu2 and Copu3, respectively. Based on the NMR data, we designated Copu2 as a new β-copaene synthase. Conversely, Copu3 was designated as a new, highly selective cubebol synthase.

**Fig. 3:**
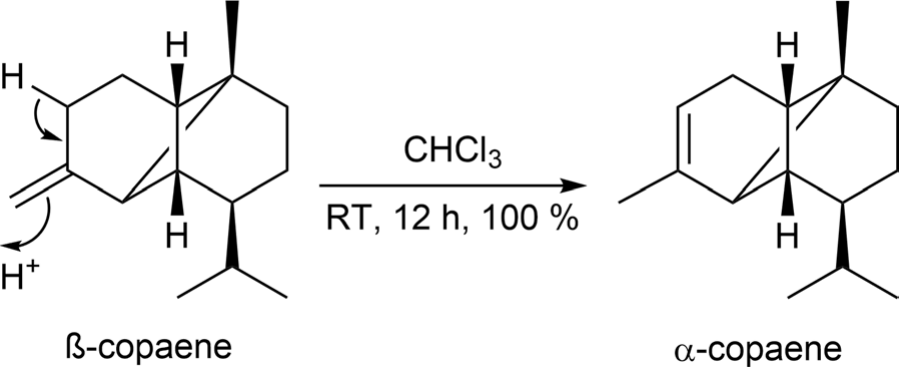
Reaction scheme to illustrate the rearrangement from β-copaene to α-copaene. This reaction is observed in the presence of chlorophorm

Interestingly, a rearrangement of β-copaene to the better described α-isomer could be observed in this context. The complete conversion took place by storing the pure compound in chloroform for < 12 h (Fig. 3). α-Copaene has been shown to possess important biological properties, including anticarcinogenic as well as antioxidant activity in the field of neurodegenerative diseases [30, 31], or serves as an insect attractant [32].

### Phylogenetic synthase evolution

**Fig. 4:**
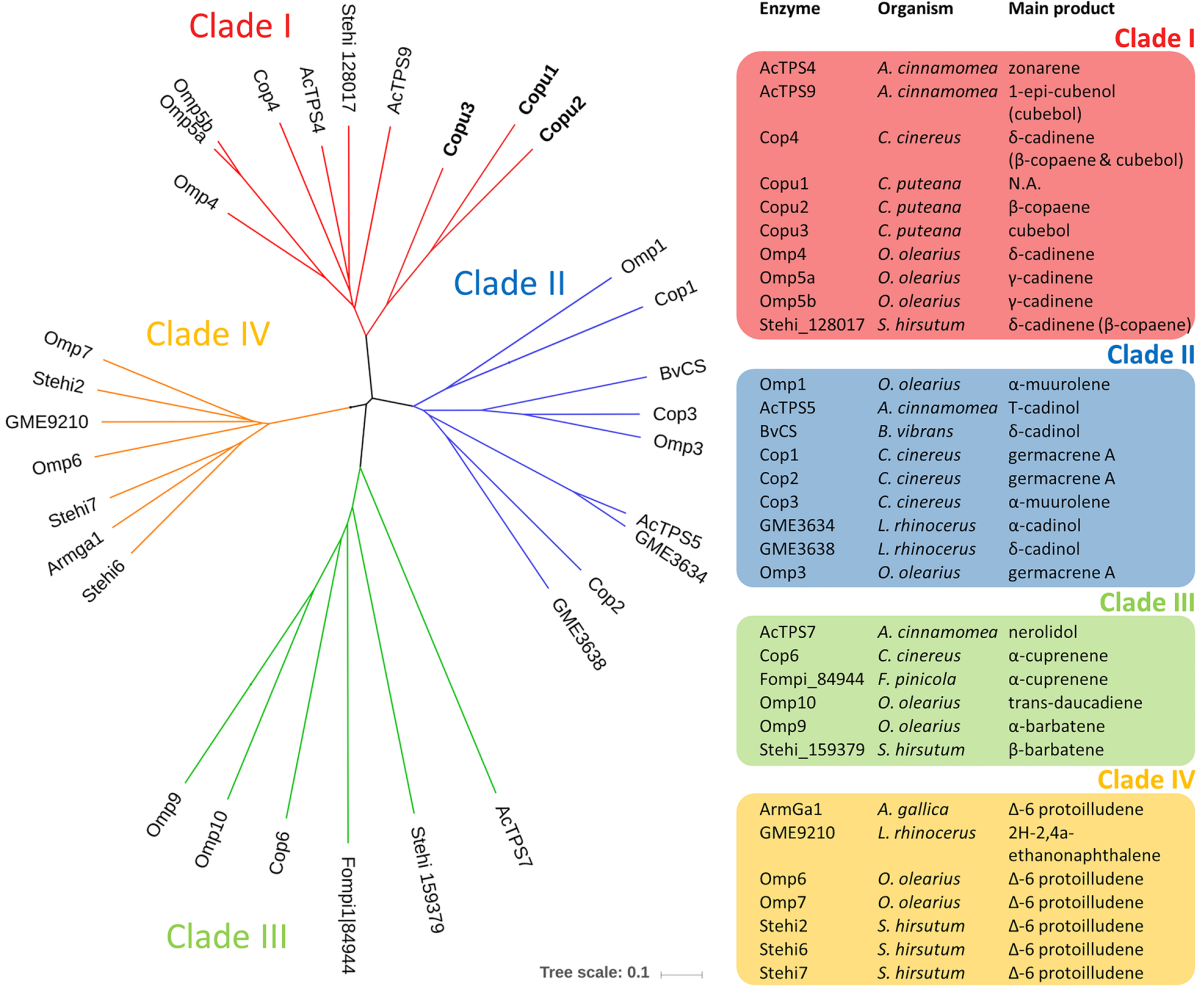
Unrooted neighbor-joining phylogram of all 29 reported and experimentally characterized STPSs from Basidiomycota. The protein sequence based analysis includes STPSs from *Antrodia cinnamomea* [39], *Armillaria gallica* [40], *Boreostereum vibrans* [41], *Coprinopsis cinereus* [35], *Fomitopsis pinicola* [42], *Lignosus rhinocerus* [43] *Stereum hirsutum* [37], *Omphalotus olearius* [42] as well as *Coniophora puteana*. The phylogenetic analysis revealed four distinct clades, divided into different cyclization mechanisms and product profiles. Copu1-3 belong to clade I including all other known β-copaene or cubebol synthases from Basidiomycota. For detailed information about the used sequences and accession numbers, see Additional file 1

**Fig. 5:**
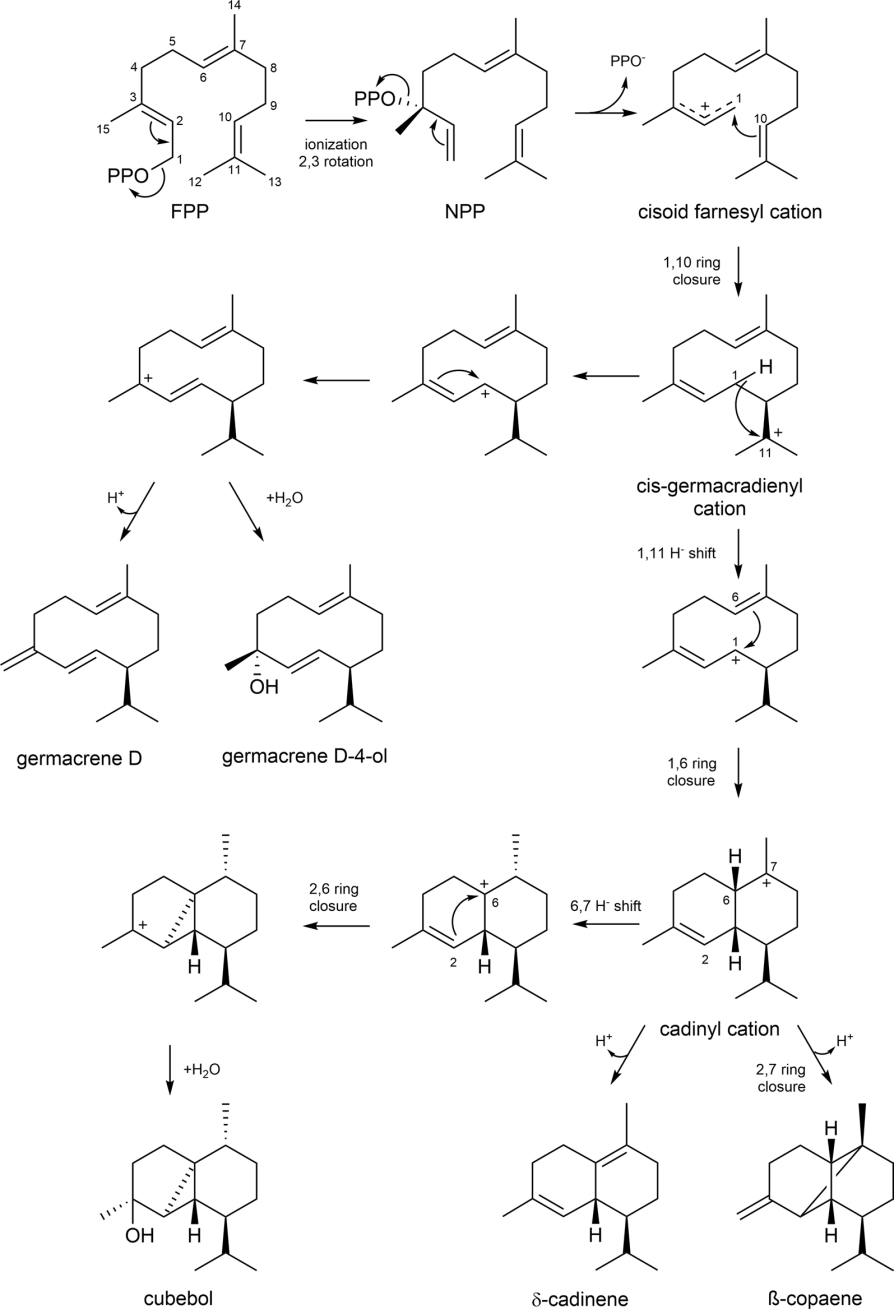
Proposed cyclization mechanisms for the generation of the main STPs from Copu2 and Copu3. The reaction starts by ionization and isomerization of farnesyl diphosphate (FPP) creating nerolidyl diphosphate (NPP). The first cyclization is achieved by the formation of the C1–C10 bond. Subsequent C6–C1 bond formation yields a cadinyl cation, which represents a general precursor for various cadalane and cubebane-type STPs [14, 44–47]

At present, the identification and characterization of terpene cyclases from the group of Basidiomycota is limited. Protein sequence-based phylogenetic analysis of the 29 genetically and biochemically characterized STPSs derived from Basidiomycota revealed four distinct clades (clade I–IV) (Fig. 4). The clustering by sequence conservation suggests that STPSs within one specific clade may catalyze the same or a related cyclization reaction. It also revealed that Copu2 and Copu3 clustered in clade I together with all other Basidiomycota-derived STPSs, generating either β-copaene or cubebol (ACTPS9, Cop4 and Stehi_128017). Additionally, most enzymes that generated cadinene isoforms clustered in clade I. For several candidate enzymes constituting this clade (Cop4, Omp4, Omp5a and 5b), a substrate cyclization mechanism has already been postulated, involving a 1,10-cyclization of (3R) nerolidyl diphosphate (NPP). The conversion of initial FPP involves the formation of a cis-germacradienyl cation, followed by a subsequent 1,6-cyclization. The final result is various STPs derived from a cadinyl cation (Fig. 5) [14, 33, 34]. By contrast, the clade II STPS mechanism involves a 1,10-cyclization of (2E,6E)-FPP to an E,E-germacradienyl cation (Omp1–3, Cop 1–3) [35], generating predominantly α-muurolene and germacrene A as well as different types of cadinol. Clade III STPSs share a 1,6-cyclization mechanism of (3R)-NPP or (3S)-NPP, leading to a bisabolyl cation [33, 36], forming mainly barbatene or α-cuprenene. Finally, clade IV STPSs share a common 1,11-cyclization mechanism of (2E,6E)-FPP [37]. Except for GME9210, all enzymes in this clade exclusively represent functionally characterized Δ6-protoilludene synthases.

In silico homology modeling [38] of Copu1-3 as well as Cop4 and structural alignments with the selinadiene cyclase (4OKM), a monomeric STPS from *Streptomyces pristinaespiralis*, as a nearest neighbor reference structure were carried out (see Additional file 1). Interestingly, the sequence identity analyses strictly differentiates sequences from distinct organisms while the structural analysis (RMSD calculation) reveals a close structural relation between Copu3 and Cop4. Both enzymes produce δ-cadinene, β-copaene and cubebol.

### Technical scale, fed-batch production of β-copaene and cubebol

**Fig. 6:**
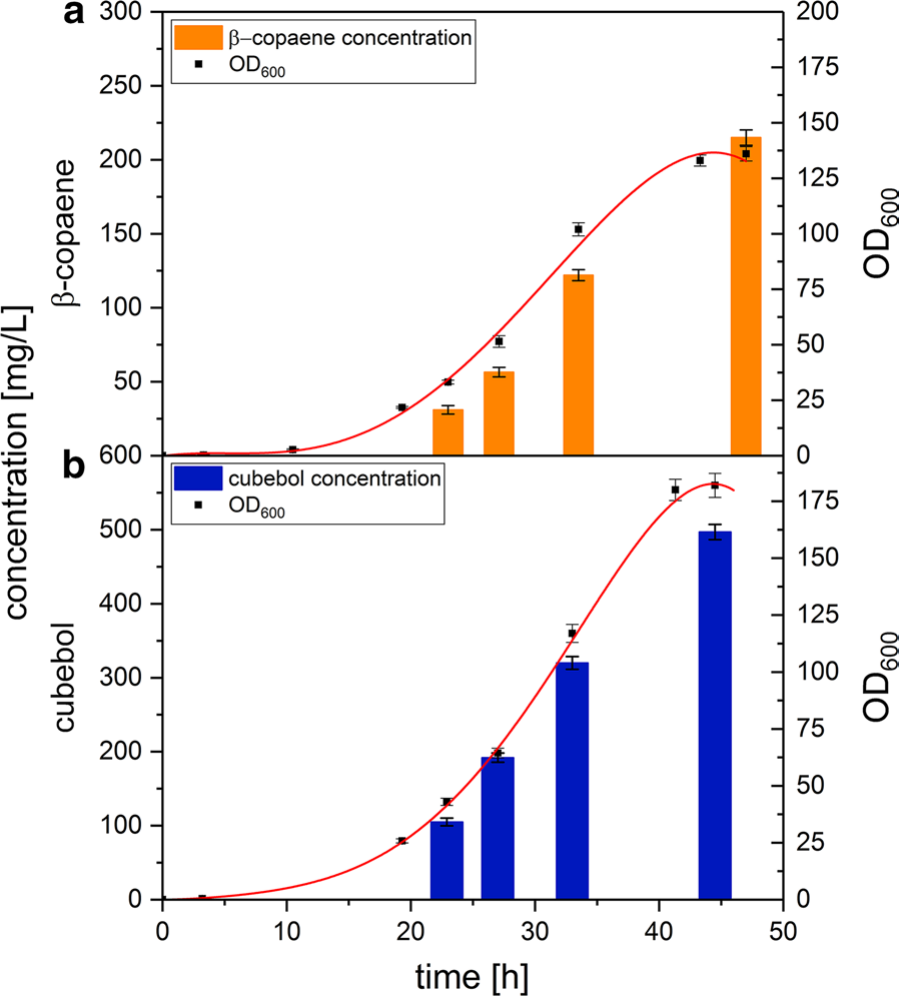
*E. coli* growth curves and time-dependent concentration of β-copaene and cubebol in controlled fed-batch fermentations. Expression of Copu2 (**a**) and Copu3 (**b**) in *E. coli* resulted in a constant concentration increase, which was finally limited by the stationary phase. The error bars represent the mean values ± standard deviation of technical triplicates

To investigate the production performance of Copu2 and Copu3 in an optimized microbial system, fed-batch fermentation experiments were carried out in 1.3-L fermenters. This production scale provided for technically relevant amounts of β-copaene and cubebol. *E. coli* HMS174 (DE3) strains co-expressing either Copu2 or Copu3 and the corresponding MEP bottleneck enzymes were cultured at 30 °C under controlled conditions (Fig. 6). The Copu2-expressing culture reached its stationary phase after 46 h with a final calculated OD_600_ of 130, providing a final β-copaene titer of 215 mg/L. Based on these data, the specific β-copaene production and productivity were 4.4 mg/g dry cell weight (DCW) and 4.7 mg/L/h, respectively. To the authors’ knowledge, this is the first report of any quantitative biotechnological production of β-copaene. In comparison, the Copu3-based fermentation generated a cubebol titer of 497 mg/L (calculated OD_600_ of 182), and the specific cubebol production and productivity even reached 7.2 mg/g DCW and 11.2 mg/L/h, respectively. The reported titers in this study exceeded concentrations of alternative approaches obtained by equivalent fermentations based on a plant-derived cubebol synthase (titers of 10 mg/L cubebol) [48] by 50-fold.

## Discussion

In this study, three new terpene synthases (Copu1-3) were identified from genome data of the Basidiomycota *Coniophora puteana* and functionally characterized for their product profiles. At present, this filamentous fungus has only been described in the context of cellulytic activity. Therefore, this is the first report of any enzyme system involved in secondary metabolite biosynthesis from this organism. Only a few reports describe the successful functional characterization of TPSs from the phylum of Basidiomycota [35, 37, 39, 42]. In this study, the heterologous expression of Copu2 and Copu3 functionally confirmed their identities as sesquiterpene synthases (STPSs). By contrast, Copu1 expression in either an engineered sesqui- or diterpene *E. coli* production system did not yield any products. The Copu1 sequence may represent a non-functional TPS variant. This could be attributed to the fact that Basidiomycota tends to have very intron-rich genomes, and various functional transcripts may be generated by alternative splicing events. The in silico identification of the annotated Copu1 coding sequence may only encompass splicing events that result in a catalytically inactive enzyme [14, 49].

The selectivity of terpene synthases varies significantly in this enzyme class. In that respect, highly selective TPSs only provide a single product (e.g., the (+)-d-cadinene synthase from *Gossypium arboreum* [46]), while more promiscuous enzymes can generate in excess of 50 different compounds (e.g., the γ-humulene synthase (*Abies grandis*) [45]). Product promiscuity is prevalent, especially within the group of STPSs [50]. However, heterologously expressed Copu2 and Copu3 display a fairly restricted product profile. As β-copaene accounted for 62% of all terpenoid products in Copu2 fermentations, this enzyme was identified as a β-copaene synthase. By contrast, cubebol was the main product (75%) of Copu3 fermentations, which designated this enzyme as a cubebol synthase. To the best of our knowledge, this is the first report of a selective β-copaene synthase and cubebol synthase. Both enzymes combine high product titers with significant product selectivity. Although we cannot completely rule out an *E. coli*-specific effect on the generated products, we believe that the enzymatic reaction corresponds to the natural conditions in *C. puteana*.

To provide insights into the structural and functional diversity of STPSs from Basidiomycota, we carried out a comprehensive phylogenetic analysis. This analysis encompassed the STPS sequences of *Antrodia cinnamomea* [39], *Armillaria gallica* [40], *Boreostereum vibrans* [41], *Coprinopsis cinereus* [35], *Fomitopsis pinicola* [42], *Lignosus rhinocerus* [43] *Stereum hirsutum* [37] and *Omphalotus olearius* [42], which are all functionally characterized. The phylogenetic analysis suggests that Copu1-3 belong to the same STPS clade, sharing a 1,10-cyclization mechanism with other related enzymes in this group (Fig. 4). Clade I encompasses all previously reported Basidiomycota STPSs that are capable of generating β-copaene, cubebol or cadinenes, which are all derived from a cadinyl cation intermediate (Fig. 5). However, Copu2 and Copu3 are the only STPSs realizing the generation of significant amounts of β-copaene and cubebol, respectively. Both enzymes form germacrene D and germacrene D-4-ol as minor products, which are derived from an earlier cis-germacradienyl cation intermediate. This carbocation intermediate is present in the biosynthesis of various cadinene type compounds [51]. The phylogenetic analysis based on amino acid sequences clearly illustrates the potential for possibly predicting enzymatic reaction mechanisms and an eventual product profile. Phylogenetic tree generation based on structural information might further improve this functional in silico prediction of an unknown terpene synthase. However, for a more precise prediction of enzymatic functions, more data have to be generated for Basidiomycota since this research area is still at a very early stage and comprises only a few candidate enzymes.

Cubebol is of interest to the cosmetic and flavor industry as it is already a registered product with a pronounced cooling effect [21] and is therefore formulated into dietary supplements and flavoring agents [9, 20]. Also the performed conversion of β-copaene to the more extensively investigated α-isoform opens up access to a potential pharmaceutical market, based on the proposed anticarcinogenic as well as antioxidant activity in the field of neurodegenerative diseases [30, 31]. At present, both compounds are commercially extracted from plant material. Specifically, β-copaene is a component of the essential oil (15%) from *Piper nemorense* leaves [52]. Similarly, cubebol is a constituent in the berry oil (5–10%) of *Piper cubeba* [53, 54] or the volatile leaf oil (3.3%) extracted from *Juniperus convallium* [55]. The quality and quantity of the target compounds from plant extracts vary seasonally and with global production location [53]. Consequently, purification from these natural extracts is technically challenging and cost-intensive [11, 12] because of various isoforms and structurally related impurities in complex mixtures. In contrast to the plant extracts, the biotechnological production of both β-copaene and cubebol would provide an efficient and sustainable alternative with simplified purification procedures. This could ensure a constant product quality, which is of high interest to the flavor industry [12].

The biotechnological formation of cubebol or β-copaene could only be provided by very few characterized STPSs. The δ-cadinene synthase VvPNCuCad (HM807407.1) from grapevine (*Vitis vinifera*) encoding a multi-product STPS shows 20.5% cubebol selectivity when expressed in *E. coli* [56]. The fungal δ-cadinene synthase Cop4 (A8NU13.1) from *C. cinereus* is also designated as a cubebol and β-copaene synthase. The product profile of recombinant Cop4 is reported to generate 30% β-copaene and 10% cubebol with respect to the total terpene production titer [35]. Even under optimized in vitro conditions, Cop4 does not generate in excess of 34.2% cubebol [47]. Other fungal STPSs with related enzymatic activity have been cloned and characterized from *A. cinnamomea* (AcTPS9) [39] and *S. hirsutum* (Stehi_128017) [37] but with significantly lower production rates. Even though several other plant enzymes are listed for the more common α-copaene isoform [57, 58], Cop4 represented the only known enzyme with a relevant formation of β-copaene to date. Therefore, Copu2 and 3 isolated from *C. puteana* are the only enzymes capable of the highly selective generation of their main products β-copaene (62% product selectivity) and cubebol (75% product selectivity) and represent an excellent basis for biotechnological production.

The only described biotechnological production approach reported for the quantitative generation of cubebol utilized a patented plant enzyme (CQ813505.1 from grapefruit *Citrus x paradisi*; 28% cubebol selectivity) but only provided titers of 10 mg/L [48], which is well below the titers we report with Copu3 fermentations. In our *E. coli*-based approach, we were able to demonstrate cubebol titers of 497 mg/L even without significant optimization of the fermentation procedures. The production of 215 mg/L β-copaene represents the first biotechnological process with technically relevant titers of this compound. Our data expand the set of functionally characterized STPSs, which can be used for biotechnological processes. With respect to literature data and current state-of-the art technology of essential oil extraction [59, 60], our production system provided, within < 2 days, cubebol concentrations (w/w) that were similar to the natural producer (ripe berries of *piper cubeba*) [53] over a whole season. At the same time, it represents a 50-fold increase compared to the only reported target-oriented biotechnological approach in yeast [48].

## Conclusion

Fungi have an enormous capacity for biosynthesis of versatile natural terpenoids and therefore represent an outstanding resource of new metabolic pathways for biotechnological production. Regarding the increasing availability of complete fungal genomes, the quantity of genetic information is expanding continuously. However, the complex biochemical characterization of the in silico-annotated enzyme activities is lagging far behind. While most enzyme-focused studies involve Ascomycota-derived sequences, the biosynthetic diversity of secondary metabolites, particularly related to the terpenome of Basidiomycota, is largely unexplored.

In this study, we focused on the discovery and investigation of sesquiterpene synthases (STPSs) from the Basidiomycota *Coniophora puteana*. We identified the STPSs Copu1-3, which were subsequently expressed in an engineered *E.* *coli* host capable of either sesqui- or diterpene production. While the expression of Copu1 did not show any terpene accumulation, the sesquiterpene β-copaene was the main product of Copu2. Hence, Copu2 was designated as the first exclusive β-copaene synthase (62% product selectivity). Similarly, Copu3 was identified as the most efficient cubebol synthase (75% product selectivity) to date. The metabolic optimization of a microbial production host, including the introduction of MEP pathway enzymes and the fungal enzymes Copu2 and Copu3, created microbial cell factories for the de novo production of 215 mg/L β-copaene and 497 mg/L cubebol. Although further work is needed to optimize the product titers, the current whole-cell systems could serve as a promising basis for the development of large-scale biotechnological production of these compounds. Patent filed.

## Methods

### Gene cloning, plasmid construction and culture condition

*Escherichia coli* strain DH5α was used for cloning and *E. coli* strain HMS174 (DE3) for terpene production. Genes encoding for the sesquiterpene synthases Copu1 (327 AA; XP_007772164.1); Copu2 (340 AA; XP_007771895.1) and Copu3 (332 AA; XP_007765978.1) from *Coniophora puteana* were ordered codon-optimized (Eurofins Genomics) for improved efficiency in *E. coli* and cloned into a pACYC-based expression vector system. The final production construct contained a single operon with selected bottleneck enzymes of the MEP pathway (DXS; 1-deoxy-d-xylulose-5-phosphate synthase from *E. coli*; WP_099145004.1) (idi; isopentenyl pyrophosphate: dimethylallyl pyrophosphate isomerase from *Haematococcus lacustris*; AAC32208.1) and the corresponding cyclase under the control of a lac-I-derived constitutive promoter [61]. Cultures were grown in modified R-media [62] (13.3 g/L KH_2_PO_4_, 4.0 g/L (NH_4_)_2_HPO_4_, 1.7 g/L citric acid, 5.0 g/L yeast extract, 35 g/L glycerol, 4.9 mL/L 1 M MgSO_4_, 2.45 mL/L 0.1 M Fe(III) citrate, 10 mL/L 100 × trace elements solution (5 g/L EDTA, 0.83 g/L FeCl_3_–6H_2_O, 84 mg/L ZnCl_2_, 13 mg/L CuCl_2_–2H_2_O, 10 mg/L CoCl_2_–2H_2_O, 10 mg/L H_3_BO_3_, 1.6 mg/L MnCl_2_–4H_2_O), 1 mg/L Thiamin) supplemented with the appropriate antibiotics, ampicillin (100 μg/mL) or chloramphenicol (34 μg/mL), at 30 °C and 90 rpm shaking.

### Terpene isolation

For analytical terpene isolation within the screening process, 35 mL of the selected cultures was mixed with 15 mL of an extraction solution (ethyl acetate, hexane and ethanol; 1:1:1). The suspension was then strongly shaken for 15 min followed by a 60 s centrifugation step at 8000*g* for phase separation. A defined volume of the upper organic phase was then sampled and analyzed by GC–MS.

### Fermentation and preparative extraction

All fermentations were performed in a DASGIP^®^ 1.3 L parallel reactor system (Eppendorf AG) using modified R-media [62] supplemented with 35 g/L glycerol. An overnight preculture was used for inoculation (OD = 0.1). The cultivation temperature was kept constant at 30 °C. The initial stirrer velocity and airflow were set to 200 rpm and 0.5 volumes of air per volume of medium per minute (vvm), respectively. The dissolved oxygen (DO) was kept at 30% and controlled by stirrer velocity (up to 1000 rpm), oxygen content (up to 100%) and airflow. A pH value of 7.00 was maintained by adding 25% sodium hydroxide solution as needed. A pH-based feed was activated by pH values exceeding 7.05, which triggered a feed shot of 40 mL. The feed solution consisted of 600 g/L glycerol, 20 g/L MgSO_4_·7H_2_O, 15 mL/L 100 × trace elements solution (5 g/L EDTA, 0.83 g/L FeCl_3_–6H_2_O, 84 mg/L ZnCl_2_, 13 mg/L CuCl_2_–2H_2_O, 10 mg/L CoCl_2_–2H_2_O, 10 mg/L H_3_BO_3_, 1.6 mg/L MnCl_2_–4H_2_O), 70 g/L collagen hydrolysate and 7.5 g/L yeast extract (pH = 7.00). Samples were taken regularly to measure the OD_600_ and the respective terpene concentration.

The whole cultivation broth was extracted by adding an equal volume of ethyl acetate and ethanol (1:1) to the cell culture. The suspension was shaken for 12 h at 22 °C followed by a centrifugation step for 15 min at 7000*g*. After separating the supernatant from the pellet, an additional 1/4 volume of hexane was added. The mixture was shaken for 2 h before the upper phase was isolated using a separation funnel. The organic layer was concentrated using a rotary evaporator. The remaining crude oil containing terpenoids was dissolved in an ACN and H_2_O (9:1) solution for further HPLC purification.

### Terpene purification, identification and quantification

The purification was performed by preparative HPLC using a NUCLEODUR^®^ C18 HTec 250/10 mm 5 µm column (MACHEREY–NAGEL GmbH & Co. KG) and a diode array UV detector at 2.2 mL/min flow rate. The injection volume was 2 mL at a concentration of 25 mg/mL of crude extract in ACN and H_2_O (9:1). The separation was performed applying an ACN gradient starting at 90% and increasing to 100% within 10 min. This was maintained for 30 min. The oven temperature was set to 30 °C. The terpene peaks were detected at 210 nm wavelength. Fractions containing the pure product, determined by GC–MS analysis, were pooled and concentrated using a rotary evaporator.

*Escherichia coli* whole-cell conversion extracts from *C. puteana* Copu1-3 were analyzed by a Trace GC Ultra with DSQ II (Thermo Scientific). The sample was loaded by TriPlus AS onto an SGE BPX5 column (30 m, I.D. 0.25 mm, film 0.25 μm). The initial oven temperature was set at 50 °C for 2 min, increased to 320 °C at a rate of 10 °C/min, and held for 3 min. MS data were recorded at 70 eV (EI) and m/z (rel. intensity in %) as total ion current (TIC). Data were collected in full scan mode (m/z 50–650). Structural determination of terpenes was conducted by comparison to mass spectra data of the NIST Standard Reference Database. Concentrations were quantified by correlating the FID peak area to a defined α-humulene and cubebol standard of known quantity (see Additional file 1 for calibration curves).

The NMR spectra of the products were recorded in CDCl_3_ (cubebol and δ-cadinene) or C_6_D_6_ (β-copaene) with a Bruker Ascend™ 400 MHz NMR spectrometer. All chemical shifts are relative to CDCl_3_ at δ = 7.26 (1H-NMR) and CDCl_3_ at δ = 77.16 (13C-NMR) or C_6_D_6_ at δ = 7.16 (1H-NMR) and C_6_D_6_ at δ = 128.06.16 (13C-NMR) using the standard δ notation in parts per million.

### Protein modeling

The webservice RaptorX was applied for homology modeling studies (http://raptorx.uchicago.edu) [63]. Subsequently, the predicted structures (Cop4, Copu 1-3) were analyzed by Visual Molecular Dynamics (VMD) (http://www.ks.uiuc.edu/Research/vmd/) [64]. Protein structures were aligned and compared using the MultiSeq [65], and scenes rendered by Tachyon [66], both, implemented in the VMD software package. Phylogenetic trees of these five proteins were calculated using sequence identity or the rmsd value as tree generation criteria.

Structure function analyses of Copu3 were performed as previously described [38].

### Phylogenetic analysis

A multiple sequence alignment was generated with Clustal Omega (https://www.ebi.ac.uk/Tools), using seeded guide trees and HMM profile techniques. Evolutionary analyses were conducted with iTOL (https://itol.embl.de/). The phylogenetic tree was inferred using the maximum likelihood method.

## Additional file

**Additional file 1.** Details on phylogenetic analysis, sequence homology, protein structure modelling and additional NMR data are provided as additional information.

## Abbreviations

AA: amino acid
DCW: dry cell weight
DMAPP: dimethylallyl pyrophosphate
FPP: farnesyl pyrophosphate
GC–MS: gas chromatography–mass spectrometry
IPP: isopentenyl pyrophosphate
MEP: non-mevalonate pathway
NIST: National Institute of Standards and Technology
NPP: nerolidyl diphosphate
STP: sesquiterpenoid
STPS: sesquiterpene synthase
TPS: terpene synthase
