# Supplementary material for: Identification of sesquiterpene synthases from the Basidiomycota Coniophora puteana for the efficient and highly selective β-copaene and cubebol production in E. coli
Source: Microb Cell Fact. 2018 Oct 22;17:164. doi: 10.1186/s12934-018-1010-z (PMC6198442; doi:10.1186/s12934-018-1010-z)
Supplement: Supplementary file 1 — Additional file 1. Details on phylogenetic analysis, sequence homology, protein structure modelling and additional NMR data are provided as additional information. [file 12934_2018_1010_MOESM1_ESM.pdf]

Phylogenetic analysis

General overview

Table S1: Detailed information about the enzymes used in the phylogenetic analysis

Lable	Species	Accession Numbers / Peptide reference	Main Product
	Antrodia cinnamomea [1]		
AcTPS4		EIW57364	zonarene
AcTPS5		EJF64719	T-cadinol
AcTPS7		EIW53985	nerolidol
AcTPS9		EIW57365	1-epi-cubenol
	Armillaria gallica [2]		
ArmGa1		P0DL13.1	Δ-6 protoilludene
	Boreostereum vibrans [3]		
BvCS		AMW90891	δ-cadinol
	Coprinus cinereus [4]		
Cop1		XP_001832573	germacrene A
Cop2		XP_001836556	germacrene A
Cop3		XP_001832925	α-muurolene
Cop4		XP_001836356	δ-cadinene
Cop6		XP_001832549	α-cuprenene
	Coniophora puteana		
Copu1		XP_007772164	---
Copu2		XP_007771895	β-copaene
Copu3		XP_007765978	cubebol
	Fomitopsis pinicola [5]		
Fompi_84944		EPT01290	α-cuprenene
	Lignosus rhinocerus [6]		
GME3634		KX281943 / ASK39765	α-cadinol
GME3638		KX281944 / ASK39768	δ-cadinol
GME9210		KX281945	2H-2,4a-ethanonaphthalene
	Omphalotus olearius [5]		
Omp1		MUStwsD_GLEAN_10001317	α-muurolene
Omp3		MUStwsD_GLEAN_10003938	germacrene A
Omp4		MUStwsD_GLEAN_10005581	δ-cadinene
Omp5a		MUStwsD_GLEAN_10000810	γ-cadinene
Omp5b		MUStwsD_GLEAN_10000811	γ-cadinene
Omp6		MUStwsD_GLEAN_10003820	Δ-6 protoilludene
Omp7		MUStwsD_GLEAN_10000831	Δ-6 protoilludene
Omp9		MUStwsD_GLEAN_10000543	α-barbatene
Omp10		MUStwsD_GLEAN_10000292	trans-daucadiene
	Stereum hirsutum [7]		
Stehi_128017		XP_007299839	δ-cadinene
Stehi_159379		XP_007306912	β-barbatene
Stehi2		XP_007301467	Δ-6 protoilludene
Stehi6		XP_007308318	Δ-6 protoilludene
Stehi7		XP_007299456	Δ-6 protoilludene

Detailed amino acid sequences

```
>AcTPS4
MSAQQFTLPDLLAVCPLKDATNPHYAQAAAESTAWVKSYNIFDARKLAFLLQGSSELLVSHAYPYAPY
EQFRTCCDFVNLLFVVDEVSDDQNGKDARRTGEVYLNVMRYPDWDDGSALAKMTREFKQRLLAFAGPN
SYRRFLMHCDDYVNAVAREAEYRERGEVLDIDAFQTLRRENSAIRLCFGLFEFALGIDLPDFVFQDPH
FMTLYWSAADMVCWSNDVYSYNMEQAKGHTGNNIVTVLMRQKSIGLQEAADLVGAHFSALMGRFVETK
KQLPSFGAAALDDAVAKYVAAMEHWVIGNLEWSFESQRYFGAEHTRVKATRVVVLSPADEN

>AcTPS5
MAVTPAPVNGSDSTKEIILKFPDFISPIPYPLRCHPQEREVSRQSEEWLLSMANFSEKQRAKFLTLNA
GLLSGWCYIDCTFDELRVCTDFMNFLFTLDDWTDEFDTTGTRGLAECVMNTLYFPDTYKSDTAAHRLT
KSFWERMRATAGPGCQQRLLSTLDTYFQAIMQQAADRGSRNIPDLEEYILLRRDTSGCKTGFAFIEYA
ANIDLPDEVIEHPIIKAMSDSTNDLVSWANDVLSYNAEQSRGDTHNLVCVLMHQNNVDRQEAIEQAGE
LWRKTLDYYFECHKALPSWGPEIDRAVALYVQGLDDWIIANAEWSFETERYFGKEGPTVKKTRQIPLL
PQRVRA

>AcTPS7
MSAGQNNPPLNVRQILSESASWSEANESALLEPFTHIFSIPGKEIRGQMIAAFNAWLNVPTDKLAIIS
KVVSMLHTASLLVDDIEDDAQLRRGVPVAHKIYGIPQTINSANYVYFLAYQELFALRSGVGADDDARE
RLVPFNELDRVVTAELLSLHRGQGLELLWRDSLQCPTEEEYVSMVNNKTGGLFRVAVKLMMACSTTNI
TVDYVPLVNLFGVYFQIRDDYMNLQSTQYAENKGFAEDLTEGKFSFPIVHGVRADTSNRQLLNVLQKR
PTTPTLKTHAIGYLRDHTKSFTYTISVMDDLEVQIREEITRLGGNARLEKIMDTLHVERPPTSQ

>AcTPS9
MSSPSSFVLPDLHAVTPFKGSFNPHYPEAAAESSEWVNSYKVLSDKKRAFFLQGGSELLCAHAYPYAG
YQQFRTTCDFVNLLFTVDEISDDQNGKGAYETGLTFYNAMSNPAYDDGTVLCKMTKEFTARLLEHCGP
QTYRRFIKHCKDYIEAVAVEADLRERGEVLDLEAYQTLRRENSAVRFCFGLAGYALGIDLPDEVVEHP
AFMAMHLSTVDMVCWSNDLYSYNMEQAMGHTGNNVITVLMQHKGLDLQGAADYTGVHFKGLIDTFLDA
KRSLPSWGPKLDGEVAQYAMAMETWVIGNLNWSFETQRYFGHARHEIKRTRVVQLYPRRIVEESSDEE
DN

>ArmGa1
MSQRIFLPDTLANWQWPRHLNPHYAEVKKASAAWAKSFRAFQTKAQEAFDRCDFNLLASFAYPLADEA
RLRSGCDLMNLFFVIDEYSDVSTEEEVRAQKDIVMDAIRNTEKPRPAGEWIGGEVSRQFWDLAKKTAS
TQAQKRFIDTFDEYLESVVQQAADRNNSHVRGIESYLEVRRNTIGAKPSFALLEFDMQLPDESHQSSG
YQRNLRKSCIDMLCLGNDVVSYNLEQARDDDGHNIVTIAMNELRTDVAGAMIWVDEYHKQLESRFMEN
FKKVPRWGGPIDLQVARYCDGLGNWVRANDQWSFESERYFGKKGPEIIQRRWITLMPKMVSEELGPQI
VDGFHL

>BvCS
MSTASSPSLVASEIDSPHHSRTSSPSPTLSPPTSFILPDLVSHCNFPLTYHPAGDEQAAASLAWMLSF
VPHFTPKKVAAMNGLQAGELTAYCYHDCPPERLRVVDDFMNYLFHLDNISDGMMAKNTTQLADWVMNA
FEWPEKFQPTVNADGEVVEEIAAVKLARDYWSRCIQQAKPGVQQRFKSSMNMFFQAVEQQTNDRDGQV
VPDLESYIDMRRDTSGCKPVFDLIEYALGFELPEEVVDHPVIKALNQDANDLVTWSNDVFSYNVEQAR
GDTHNMICIFMEHDGCTLQEAIDRVGGLCKQTIDAFVENKARVPSFAHLGPEVDAWTTGYVQGLQDWI
VGSLHWSFMTKRYFQEAGAEVKKTRFVKLLPIEEGRHKHIPPIYASAMVAATA

>Cop1
MVNLSWYWQGQGNISKSIGPPSQTYTKSVLREQSMTFRMLALQSGLKSAASDHVSTSGSGILRFLSRI
LPANTTRRCSACCTEMSSLDATIHPVLNFEDKKIVLPDLVSHCNFKLRVSRHRKRITGETKRWLFKGD
NLVGPARNKYHGLKAGLLTAMTYPDAAYPQLRLCNDFLTYLFHIDNLSDDMDNRGTWSTANEVLNSLY
HPYTYHGQARVGRMTRDYWRRMILTASPGSQQRFIETFDFFFQSVTQQAIDRLTGEIPDLESYIALRR
DTSGCKPCWALIEYANNLDLPDEVMDHPVVRSLGEAANDLVTWSNDIFSFNVEQSKGDTHNMIPVVMH
QEGLDLQSAVDFVGEMLDHTSTCCGLESPLWLTPPLRIRRNAPRLYRVFL
```

```
>Cop2
MPSPAGALPKSFILPDLVNDCPFPLRVNPLCDEVGRLSEQWFLRHANYSPPRAVAFMALKAGELTAAC
YPDADAFHLRVSDDFMNFLFNADDWLDDFDIEDTYGLANCTVRALRDPVNFITDKRAGLMTKSYFSRF
LKTAGPRCTERFIQTLALYFESVVTQKQARNNGTLPDLESYITIRRNNSGCKPCYALIEFCAGIDLPD
EVINHPIIQSLEDASNDLIAWSNDIFSFNREQSRHDSFNMVSIVMHQKGFALQEAVNFVGELCKKAME
RFQADKRNLPSWGPEIDGEVAMYVDGLQNWIVGSLNWSIDGTERYFGKDGPGIKKHRKVKLFPKRPLK
TPAVRVLA

>Cop3
MSTPSSSLTTDESPASFILPDLVSHCPFPLRYHPKGDEVAKQTVHWLDSNCPDLTAKERKAMYGLQAG
ELTGYCYPYTTPERLRVVADFLNYLFHLDNISDGMMTRETAVLADVVMNALWFPEDYRPTKGQAAEEL
NPGKLARDFWSRCIPDCGPGTQARFKETFGSFFEAVNIQARARDEGVIPDLESYIDVRRDTSGCKPCW
VLIEYALGIDLPDFVVEHPVIAALNQGTNDLVTWSNDIFSYNVEQSKGDTHNMIIILMEHHGHTLQSA
VDYVGSLCQQTINTFCENKQQLPSWGPEIDDMVAKYVQGLEDWIVGSLHWSFQTRRYFGDEGQEIKQH
RLVKLLTVAPPPPPPPPTPPPQSSDADTKKQKVKAQDGKGPVSDEEVWALVRAEQSKGSILESLFGFL
TTSLSRIFFGYFFAYSH

>Cop4
MRPTARQFTLPDLFSICPLQDATNPWYKQAAAESRAWINSYNIFTDRKRAFFIQGSNELLCSHVYAYA
GYEQFRTCCDFVNLLFVVDEISDDQNGQDARATGRIFVNAMRDAHWDDGSILAKITHEFRERFVRLAG
PKTVRRFADLCESYTDCVAREAELRERNQVLGLNDFIALRRQNSAVLLCYSLVEYILGIDLDDEVYED
PTFAKAYWAACDFVCWANDVYSYDMEQAKGHTGNNVVTVLMKEKDLSLQEASDYIGRECEKQMRDYLE
AKSQLLQSTDLPQEAVRYIEALGYWMVGNLVWSFESQRYFGAQHERVKATHVVHLRPSSVLEASCDSD
SDSDC

>Cop6
MPAALPYNVSRDNKWDIKKIIQDFFKRCDVPYQVIPYDTELWNACLKRAKEKGYPVEPDSPMSLYRSF
KVGVVITRTSYGHIQDYEILIWVATFTAFVTYADDAFQEDIQHLHSFARTFLQNEKHEHPVLEAFAQF
LRESSIRFSHFVANTVVSSALRFMMSIALEFEGQNVSVSTEAREYPGYIRILSGLSDIYALFAFPMDL
PRSTYIQAFPEQIDYINGTNDLLSFYKEELDCETVNFISAAATSQQVSKLEVLRNAAEKAAYSYDVVV
NVLKPYPEALAAWKSFARGFCYFHTSSPRYRLGEMFHDFEHDLVCKCASCTEI

>Copu1
MGLPQATEFVLPDFFAPCPFTLGLTNPHADTVFPEARAWIGKFLPFSGLAWHCDPWAGKEGFRTICDF
QNLLFLMDELTDEMSGEDARVVGESFIRVLKDPSVDNESIIAQATREFRTRIADDVATKSVWFGRFVA
ICKSYADATYIEAEHRENNRVLDLDDFVIARRENSAVRCCFSINEHALGIDLPDSVFEDPEFLRMYFD
AVDMIVIVNDVYSYNMEQAKGLAGNNVVTVLEQALGVDLQAAVNRGGEMFAQKMEGYVRGRGVPPSWG
AKVDADVEHFFDSVDQWIVGNLEWSTETSRYLGPDHEEIMKTGRVVLRKIESKTK

>Copu2
MSTMDPSEFILPDFFATCPFAFGRTNPHADVVIPEAHAWIVKHVPFVDRKRDEFIQDGFQDLMPHCYP
WAGKETLRTMCYLNNLLFLVDDLTDDMNSDEARGFGESFIRVLNDPAVHDSSQVMQATREFRSRITGT
GVTESRWFGRFLAIFKLYINAVCAEAEDRENKRILDLDTFTVARRENSAVMVFFAITEYALGIDLPDA
VYEDPTFLRVYADSADMVILVNDVFSYNREQAKGLDGNNNITVLMQTLDLDLQAAVDHVGEMFSQKME
GCMRGRAMLPSWGVKVDADVERFFDALDQWVVGNLEWSSQSPRYLGPEHEEIMRTRRVVLRKVETEIE

>Copu3
MSATPAPTEFILPNLFSVCPLTFGRSNPYYDEVIPEARAWIAKYNPFVDSKRAEFVQGCNELLCSRVW
PYAGREEFRTCCDFVNLLFVLDELSDDMGGADARSTCDSFIRVLNDPDAPDTSLIAQMTREFRARVAE
RAKPGCLRRFIALCGTYVEAVCVEAELREQGRVLDLRSFILLRRENSAVRCCLALAEYALGLELPDAV
FNDPAFQSVYFCAADMVCWSNDVYSYNMEQAKGHTGNNVVTVLMQEHGIDLQAAADRVGEVFGQLMEH
YTSGSRSLPTWGGKVDADAARFLEAAGQWVVGNLEWSFETPRYFGPDHDEVRDTHRVLLK
```

>Fompi1|84944
MSRRYQTSRFSPSAKGKKTSLYTSPGDLRALFHRFSLYAGVQLESFTSPKMMQIEVPVMLNLKESSMP
MNSTVLEDCTAAVAKDAIVQFLRRLGAVLRPSFGNNRDLEERVKEITKTWPFEHRIHPHITTGVVMAN
TTIAYLSDLDARAAVAAYTALITALDDPDIFHASGAQNFAQMLCDGSALRDDGVLGQMARVLADMGNH
FPPFGTSAIIAATLRWCNGELISNPANPFCLRPLSKAFADYQRGLTGVPEAYAAFGWCKADFPVETDY
IHVFPDICFFLNHTNDILSFYKETLDGESDSYIHARARLTGKSVTDTLYEVMDEVITTTERIRKHFGE
GRMRNAWDRYEAGYVWFHTGNPRYRLHELVDTEYMPMY

>GME3634
MAVTPANVASPDSQEIVLKFPDFISPIPYPLRCHVQEREVSRQSEEWLLSMANFSEKQRSKFLTLNGG
LLSGMCYIDCTFDELRVCTDFMNFLFTLDDWTDEFDTTGTRGLAECVMNTLYWPHSYQADTAAHRLTK
SFWVRMKQTAGPGCQQRLMSTLDTYFQAIMQQAADRGSHNIPELEEYILLRRDTSGCKIGFAFIEYAA
NIDLPDDVIEHPIIKAMADATNDLVSWANDVLSYNAEQSRGDTHNLVCVLMAQNGLDRQGAIELAGEL
WEKTLHLFFECRKNVPSWGSEIDRAVALYIQGLEDWIIANAEWSFETERYFGKDGHLVKKTRQVTLLP
VRTAA

>GME3638
MRARSFILPDLVSDCPYTLRCNSNCEAVARASEAWMLEDANLSPKRRDAFLRLRGGELTAACYPDTDE
ACLRVAADFLNFLFSLDDWSDEFSMEDTCGLAQCVMCVLHDPDDFQTEKAAGKLAKSFFNRFRQTAGP
RCTRRFIDSMDLFFHAIAQQAQDRASGSAPSLEEYVALREDTSGCKPCFALIEYAAGMDLPDHVAHHP
TITALEREANACISWSNDLFSYNVEQARGDTHNMIAVIMREDGRSLQEAVEYLGALCKLCIVHFEENR
AMLPSWGPEIDGEVDRYVLGLQDWMVGALHWSFDTARYFGDEGPAIKKHGVVTLLPRKSSS

>GME9210
MPLSSSVVAFRLPDTLGCWPWRRCLNTHYVEAKQDSASWLESFHPFGPKAQRAFNKCDFNLLASLAYP
VASKDQLRAGCDLMNVFFVFDEYSDVENEKTVQQLADIIMDALRNPHKPRPAGESLVGEITRQFWART
IKVASEPSQRRFIETFDDYCQSVVQQAADRSQNHLRDVESYLENRRENIGAKPSFALLELDMNLPDEV
IEHPTIVNLTTWAIDMIILGNDIVSYNVEQARGDDGHNVVTIVMHHYNVDVQGAMDRIAEWHQRLADQ
FLTNYNKLPSWGREIDAQVERYIQGIGNWVRANDAWSFESERYFGLNGREIEQSRWVTLLPRVSAEKP
AVV

>Omp1
MKYTSFALPDLASSCDYNLRFNKYHRSVSPETKKWFFRLSPASQADLTTYDAQRFTLLAAVCYPDAGY
PQLRVCSDFLAYLFYLDNLTDDMDDKSTRSVADLVLNSLNEPETFQTQYRIGKMTSDYFKRIIQTSND
GTKKRFIDTMDSFFKSVDDQARDRLAGHIPDLESYIALRRETSGCKTCFSLIEYANNLHIPDEVISHP
HIEQMETAANDVVSFANDIYSFNIEQSKGDTHNMIPVLMHANPDMDFLEAVSFVRDLTIKAMDRFNEL
RATLPSWGLDIDKDMKVYVNGLENWMVGILFWSFETERYFGKSVRSVKATKTVNLLPSRA

>Omp3
MAIENTIASAPASTPAKQLDTPDHFILPDLVSHCTFPLVYHSNGDAVAAQSVKWLDTNCPDLNDKRRK
ALYGLQAGELTAYCYNTAPDQRLRVVSDFMNYLFHLDNISDGMMTKDTDALSDAVMNALWFTEWYRPT
KKSDYVQPDEELNAGKLARDFWHRCIQDAGPGCQARFKETLELFFEAVNIQAKARDAGVIPDLESYID
VRRDTSGCKPCWALIEYGLGIDLPDYVAEDPIIKSLNQSTNDLVTWSNDIFSYNVEQSRGDTHNMIVI
LMLYHGHNLQSAIDYVGDLCRQTIDDFKENRKKIPSWGPEVDDIVKQYVQGLQDWIVGSLHWSFMTTR
YFGKQGQEVKKNRYVKLLPVGEEANKW

>Omp4
MSSAPTRFLLPDLLSACPLKGSVNPYYKEAGAESSAWINSYDIFTDRKRAFFVQGCNELLVAHTYPYA
GYEEFRTCCDFINVLFVLDEVSDEQSGSDARFTGEVFLNALRNPENDDTSKLSKISKEFRARYFKRAG
PRTAERFLQHCQDYIDCVTREAELRERGEVLDLPSFTALRRENSAIRVCFCLFEYALGFDLPQEVFDD
PTFMEMYWAAADLVCWANDVYSYNKEQAQGHGGNNIVTVLMKAKDLDLQAACDYIGVYCEELMGRYLS
AKARLPSWGPEVDAAVAQYVEASGHWVRGNLDWSFETQRYFGAQHAEIKETRLVTLTPAIPEDFSDTG
SESE

```
>Omp5a
MSPDPTRIVLPDFLAACPFESSTKNPHLKAAGAESSAWVNSHVVFNDRKRAAFMQDIYELLVAYAFPW
ADYEDFRTMCDFINLLFVLDELSDDQNGKDAGYTGKLFMDAMRNIDNGDTSELTELCREFKARYSKRV
SPQVNERFLQHLQSYTDCVAQEADLRERGEILDLESYVALRRENSAIRPCFDLVEYIIDFDIPQEVID
HPVFSEMYWASVDLVCWSNDVYSYNVEQAKGHGGSNVVTVLMKEKNLDLQAACDYVGVYYEELMDRYL
SAKARLPSWGPEIDAAVGKYILAEAQFVRGNLDWSFDSPRYFGPQHDQVKKTGIVTLTPAPKKFGSDS
GSESE

>Omp5b
MSPAPSRIVLPDFFASCPFESSTINPHFKAAGAESSAWVNSHVVFNDRKRAALMQNSYELLVAYAFPW
ASYEDFRTLCDFINLLFVFDEVSDDQNGKDAGYTSKIFMDAMRNIDNGDHSELTELCKEFKARFSRRL
SPQVNERFLQHLQSYTDCVAQEADLRERGEILDLESYVILRRENSAVRPCFDLVEYIMDFDIPQEVLD
HPVFSEMYWASVDLVCWSNDVYSYNVEQAKGHRGSNVVTVLMNEKNLDLQAACDYVGVYYQELMDRYL
SAKARLPSWGPEIDAAVGKYVLAEAQFVRGNLDWSFDTPRYFGPQRDQIKKSRIVTLTPAPKKFGSDS
GSESE

>Omp6
MIAKNSEIDRFYIPDTLANWPWPRHLNPAYPEAKKASAAWLRSFNAFNERSQKAFDLCDFNLLASLAF
PLADLYCLRSGCDLMNCFFIFDEYSDVADPQTVRQQADIIMDAIRNPHVPRPRGEFIGGEAHRQFWER
AMQGATPTAQRRFIDTYQQYTDAVVQQATDRADNHIRDVEGYFTVRRDTIGAKPSFTLLEFTMDIPDE
VMGHPVIKDLSLWCIDMLIIGNDLCSYNVEQAHGDDLHNLVTIVMNQYNLDLPGAMEWIGKFHDDIAD
KFLDTFAKLPSWGPEIDPQIRRYVDGLGNWVRGNDSWSFESWRYFRGKGPEIEKTRWVDLMPTEEATI
TPKYESDSNAAQPAQST

>Omp7
MPETFYLPDCLANWKWKRALNPNYPEVKAASSEWLRSFKAFPPKAQEAYDRCDFNLLASLAYPLADKD
GLRTGCDLMNMFFVFDEYSDVAHESEVQVQADIIMDALRNPHKPRPVGEWVGGEVTRQFWELAIKTAS
PQSQKRFIETFDTYTKSVVQQAADRTQHYVRTVDEYLEVRRDTIGAKPSFAILELTMDIPDEVIHHPT
IERLAILAIDMILLGNDTASYNYEQARGDDNHNMVTIVMHQYKTDIQGALSWIEKYHKELEEEFMQLY
NSLPKWGGQIDVDIARYVDGLGNWVRASDQWGFESERYFGTKAPEIQKTRWVTLMPKKRAEGVGPEIV
DISEL

>Omp9
MSQILHLLWSKFSTSLPSTVTIGSDPQTLQLVHSPAPNVNANALEIYKIVDNFLSRCGIRLESTPLDV
EFYNECKKTLLSHYIGIHDSDKVSESWFKRYLSVGVIITTNAYGHLDNKLTKIYIALYTALATCFDDV
FEKNVDHMSGFNERFMKALPQGDVFLDAFAKVLLDAPKYFGRLASNIIVTSTLDFITSMSVDVLTKGM
KFNQNLHKFAMACRNMSGIAYTYAPFIFPKEVPFAIYAQCLPDMRIYINHVNDVLSFYKEDKAGETEN
LASILGQVHPSMTKYQIVQGLADDAAEADLRVRTVLSQYQPALDAYNCFRQGYVSFHASSGRYRLDEL
FSFVEPEPIV

>Omp10
MTLPTEQVELSVCPVESSTHTTRDIMRNFLSQCQIPLQRGVPLDPTFHQECANVLIEDYLKPSAAVTL
ENLPSLMSSFNPFLTLGVRMASTGYAHLTHTPTRVYVALFTALLVCLDDIFPENVELMCGFNERFIKN
ETQGEPILDAVAGLLRSTSKYFSMLSSNLIVTSALNYVTSLSLDQGLHSIKLAEHSRNFARLCRNMSG
IPEAFAAFVFPPEVPFTAYIQCFPDLYTYANYVNDVLSFYKEDIAGETENLVSILAQTQPNSSRYQVL
QQLADEAAAANANIRDILSDQKSILDAYDAFRVGFVQFHIDSPRYRLAELFPCIDG

>Stehi_128017
MAAPESSAWVSSYNLFSDRKRTDFITGSNELLVSHTYPHADYDAFRTCCDFVNLLFVIDEISDDQSGK
AARRTGEVYLNAMRDPEWTDGSDLAKMTQQFRARFLRSVGPQSFRRFLRHSEDYIDCVAKEAEYRERG
QVLDMDSFKSLRRENSAIRLCFGLFEFTLGIDLPDSVFEDETFMKMYWASADMVCWANDVYSYNVEQA
KGHSGNNIVTVLMAARDIDMQAASDYVGEYYAELMEEYMTAKAELASKSFGSRDLDEDVWKYVNAMEN
WPIGNLEWSFKTNRYFGTLHDEVKRTRLVVIKPRKVVV
```

```
>Stehi_159379
MAHPTTNPSHRLEQMESVREHIPRLQHFLGEIGYRHTTPPAPTLDFLHAHHHWIHHVLGPMTSWTVAK
LNALEDSSSTIFERAYPLSDAEMKFVLAKLTAIAIFLDDSLEDEETYDDIGNFAHRVYLGEAQPTGVL
TLYHQGIQELSKMHEGDAVFRGLAVAPWITFIDACMLEKRLLTFDSKLRVSPRDLGYQRLRNSTDFTS
LRAPKATPSEVEVSFPIFLRHKSGIGEAYAAAIFKSSRYQELPLSRFVKSMPDMIYYIELVNDLMSFY
KEQLAGETANLIHLQHQSWKGGQGTGPYGSWTLLDTFSRLCDETRDAAFRVDELLRLDECEKIANGEL
RGEEVGLSPMDVTMAAQWREFRDGYVSWHLECQRYKLDFIKLSTFE

>Stehi2
PRHINPHYQEVKKASAAWAESFGAFNPKAQHAYNACDFKRLRTGCDLMNMFFVFDEYSDVSSPKDVIQ
QAAIIMDALRNPYAPRPDDEWVGGEVTRQFWKRAIKTATAGAQRRFIDAFESYTQSVVQQAKDRHHGF
IRDVDSYLEMRRETIGAKPSFVVLQMDMTLPDEVLAHPVIQQLSALSTDMICLGNRRLIQILWTVQDI
CSYNVEQARGDDLHNIITIAMNQFDIDIAGAMDWVVKYHAKLERKFLYLYNNGLPSWGKELDPQVERY
VCGL

>Stehi6
MVRSPVSDKFCIPDTLASWPYPRILNPHYAEEKAASAAWTKGFGAFGPKAQDAFDRCDFKRCRSGCDL
MNLFFVIDEHSDTHGEETVRKMKDVVMDAIRNPHKPRPNDEWIGGEIARQFWERAMCYASEISQRRFI
DTFDEYLESVVDQAADRDSARIRDIESYINIRRNTIGAKPSFVIMEQGMDIPDNVFENEVFQRLRMAT
IDMLCLGNDIVSYNIEQARGDDSHNIVRIVMNELDTDVPRAMDWVAQRHTQLEREFFTALSELPTWGE
PIDGWVKEYVYGLGNWVRANDQWSFESQRYFGTKGMEIMKSRWLSVLPKVRPAEVGPQLVDQSLL

>Stehi7
MAVATSVATPVPTPAYSAGRAPAKEKKIYLPDTLAEWPWPRAINPHYAEAKEESQAWAASFNAFSPKA
QHAFNRCDFNLLASLAYPLATKHGCRSGCDLMNLFFVIDEYSDIAPVEEVRQQKDIVMDALRNPHKPR
PEGEWVGGEVARQFWALTITNASAQSQKHFIETFDEYLDSVVQQAEDRSESRIRDIQSYIDVRRNTIG
AKPSFALLELDMDLPDEVLAHPTIQSLSLATIDMLCLGNDIVSYNLEQARGDASHNIITIVMNELNLD
VNGAMRWVGDFHKQLEKQFFEAFNNLPKWGNAELDAQIAVYCDGLGNWVRANDQWSFESERYFGARGL
EIMETKTLAMMPIQRTEALGPQLVDDSIL
```

Sequence analysis

```
Cop4          --MRPTARQFTLPDLFSICPLQ-DATNPWYKQAAAESRAWINSYNIFTDRKRAFFIQGSN 57
Copu1         -MGLPQATEFVLPDFFAPCPFTLGLTNPHADTVFPEARAWIGKFLPFS------------ 47
Copu2         -MSTMDPSEFILPDFFATCPFAFGRTNPHADVVIPEAHAWIVKHVPFVDRKRDEFIQDGF 59
Copu3         MSATPAPTEFILPNLFSVCPLTFGRSNPYYDEVIPEARAWIAKYNPFVDSKRAEFVQGCN 60
                      :* **::*: **:  . :**  . .  *::*** ..  *
                                         D(D/E)xxD
Cop4          ELLCSHVYAYAGYEQFRTCCDFVNLLFVVDEISDDQNGQDARATGRIFVNAMRDAHWDDG 117
Copu1         -GLAWHCDPWAGKEGFRTICDFQNLLFLMDELTDEMSGEDARVVGESFIRVLKDPSVDNE 106
Copu2         QDLMPHCYPWAGKETLRTMCYLNNLLFLVDDLTDDMNSDEARGFGESFIRVLNDPAVHDS 119
Copu3         ELLCSRVWPYAGREEFRTCCDFVNLLFVLDELSDDMGGADARSTCDSFIRVLNDPDAPDT 120
                *  :   :** * :** * : ****::*:::*: .. :**     *:..:.*    :

Cop4          SILAKITHEFRERFVRLA--GPKTVRRFADLCESYTDCVAREAELRERNQVLGLNDFIAL 175
Copu1         SIIAQATREFRTRIADDVATKSVWFGRFVAICKSYADATYIEAEHRENNRVLDLDDFVIA 166
Copu2         SQVMQATREFRSRITGTGVTESRWFGRFLAIFKLYINAVCAEAEDRENKRILDLDTFTVA 179
Copu3         SLIAQMTREFRARVAERA--KPGCLRRFIALCGTYVEAVCVEAELREQGRVLDLRSFILL 178
              * : : *:*** *..         . **  :   * :..  *** **. ::*.*  *
   Pyrophosphate sensor                                   (N/D)Dxx(S/T)xxxE (NSE)
Cop4          RRQNSAVLLCYSLVEYILGIDLDDEVYEDPTFAKAYWAACDFVCWANDVYSYDMEQAKGH 235
Copu1         RRENSAVRCCFSINEHALGIDLPDSVFEDPEFLRMYFDAVDMIVIVNDVYSYNMEQAKGL 226
Copu2         RRENSAVMVFFAITEYALGIDLPDAVYEDPTFLRVYADSADMVILVNDVFSYNREQAKGL 239
Copu3         RRENSAVRCCLALAEYALGLELPDAVFNDPAFQSVYFCAADMVCWSNDVYSYNMEQAKGH 238
              **:****    :: *: **::* * *::** *   *  : *::   ***:**: *****

Cop4          TGNNVVTVLMKEKDLSLQEASDYIGRECEKQMRDYLEAKSQLLQ-STDLPQEAVRYIEAL 294
Copu1         AGNNVVTVLEQALGVDLQAAVNRGGEMFAQKMEGYVRGRGVPPSWGAKVDADVEHFFDSV 286
Copu2         DGNNNITVLMQTLDLDLQAAVDHVGEMFSQKMEGCMRGRAMLPSWGVKVDADVERFFDAL 299
Copu3         TGNNVVTVLMQEHGIDLQAAADRVGEVFGQLMEHYTSGSRSLPTWGGKVDADAARFLEAA 298
               *** :*** :  .:.** * :  *.   : *.    .       . .:  :. :::::

                            RY-dimer
Cop4          GYWMVGNLVWSFESQRYFGAQHERVKATHVVHLRPSSVLEASCDSDSDSDC          345
Copu1         DQWIVGNLEWSTETSRYLGPDHEEIMKTGRVVLRKIESKTK----------          327
Copu2         DQWVVGNLEWSSQSPRYLGPEHEEIMRTRRVVLRKVETEIE----------          340
Copu3         GQWVVGNLEWSFETPRYFGPDHDEVRDTHRVLLK-----------------          332
              . *:**** ** :: **:* :*:.:  *  * *:
```

***Figure S1**: Alignment of Copu1-3 and Cop4 (as reference) amino acid protein sequences. Typical signature motifs for class I terpene cyclases such as the highly conserved NSE-triad and the aspartate-rich D(D/E)xxD motif are highlighted. Additionally, a highly conserved arginine residue 46 AA downstream of the NSE motif, which is indicated as pyrophosphate sensor and a common conserved RY-dimer ~80 AA downstream of the NSE motif, close to the C-terminus is found in all four sequences [8–11].*

Enzyme modelling

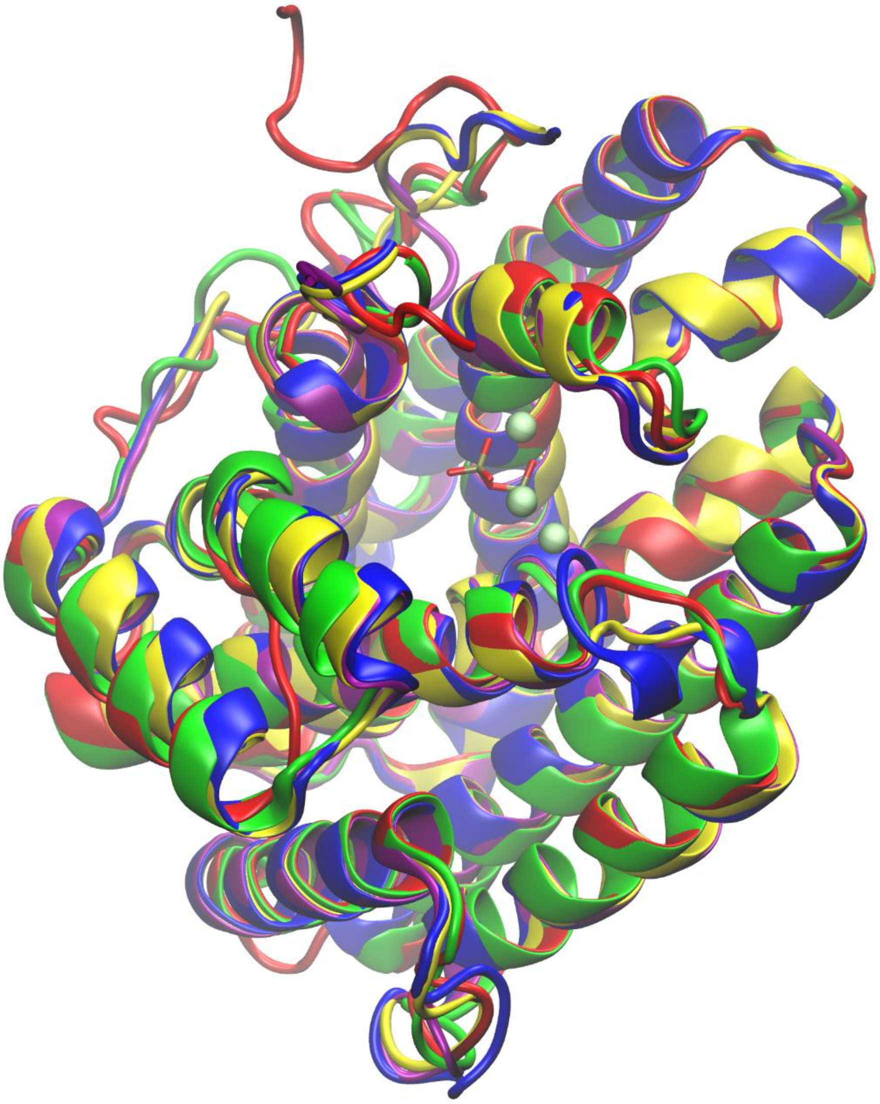

Figure S2*: Homology modeling and structural alignment of selinadiene cyclase (blue), Copu1 (red), Copu2 (green), Copu3 (yellow) and Cop4 (purple).*

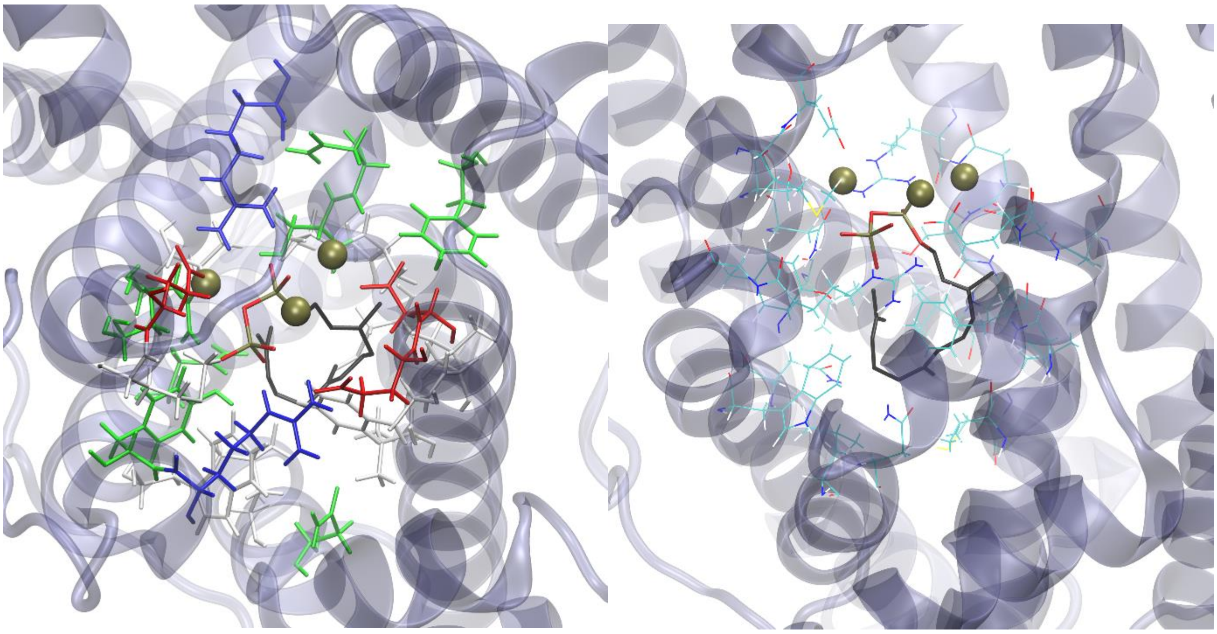

Figure S3*: Structure function analyses of copu3 revealed reliable positioning of FPP in the active sites cleft. Amino acid sidechains in close proximity (5 Angstrom) to the docked FPP molecules (black) are shown. Sidechains are colored based on their physico-chemical characteristics (hydrophobic - white, alkaline - blue, acidic -red, polar - green).*

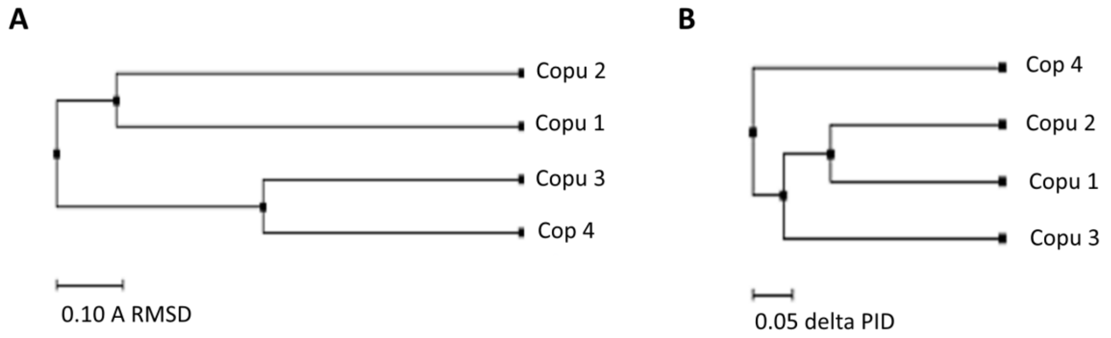

Figure S4*: Structural identity analysis (RMSD calculation) (****A****) and sequence identity analyses (****B****) of the Copu1-3 and Cop4 sequences. Sequence identity analysis strictly differentiate sequences from distinct organisms while the structural analysis (RMSD calculation) revealed a close structural relation between Cop4 and Copu3.*

Structure analysis and quantification

NMR spectra

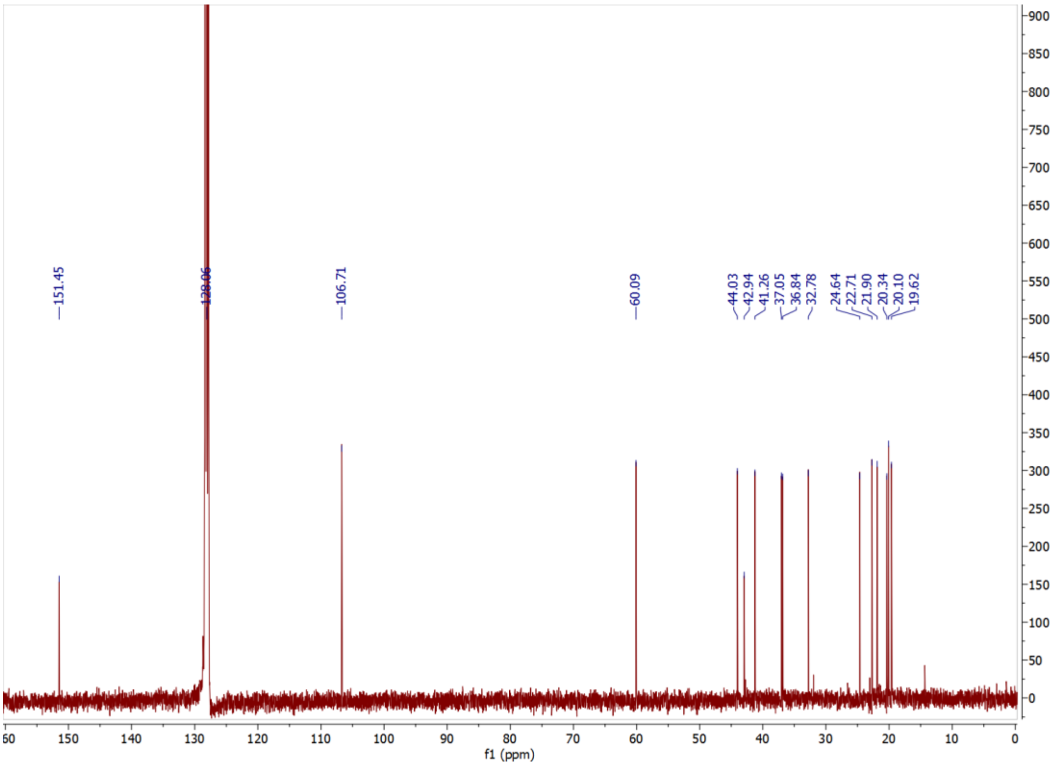

Figure S5: *β-Copaene ^{13}C NMR data recorded in C_6D_6.*

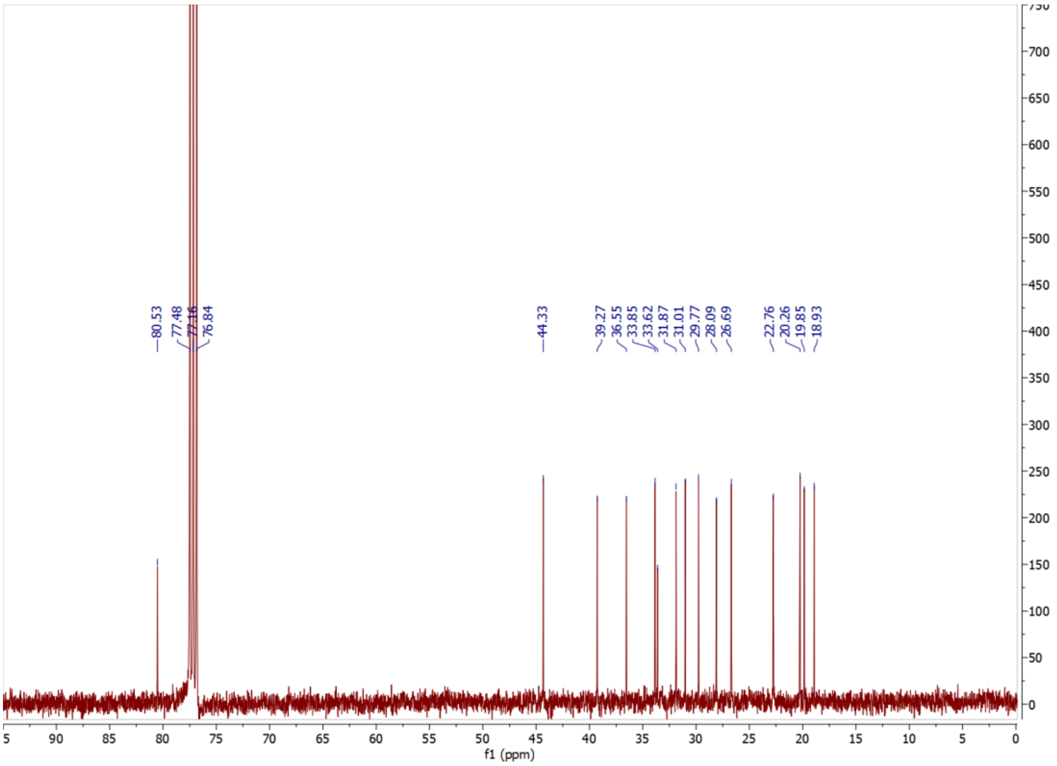

Figure S6*: Cubebol ^{13}C NMR data recorded in $CDCl_3$.*

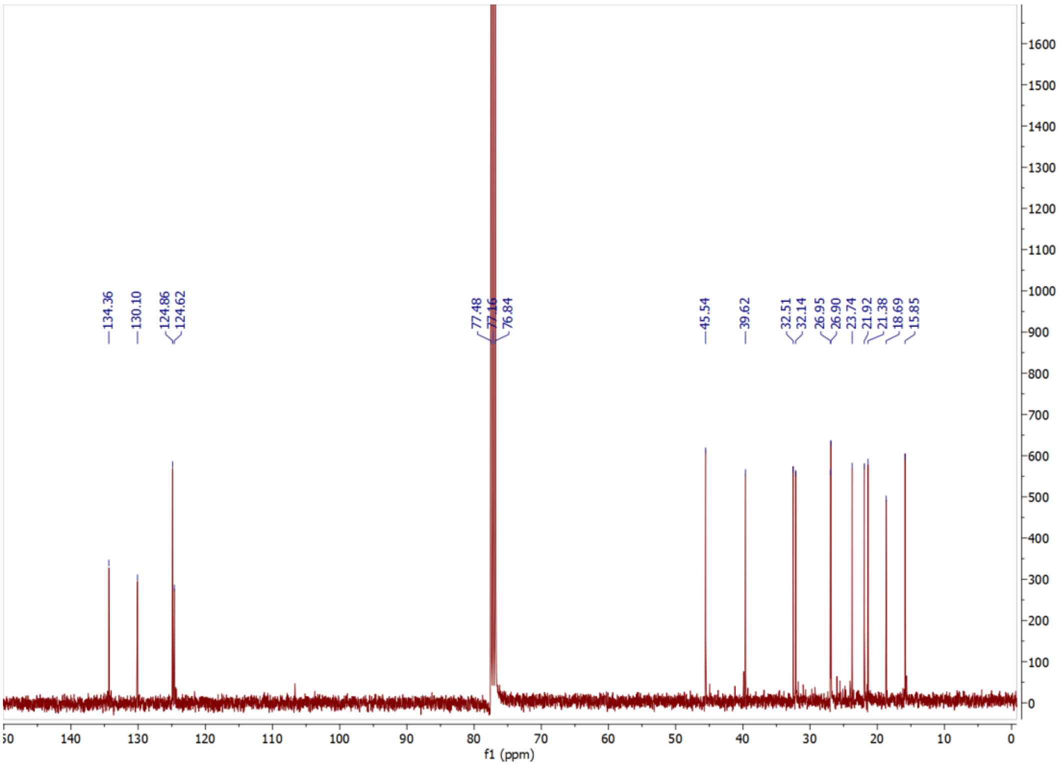

Figure S7: *δ-Cadinene ^{13}C NMR data recorded in $CDCl_3$.*

Terpene quantification

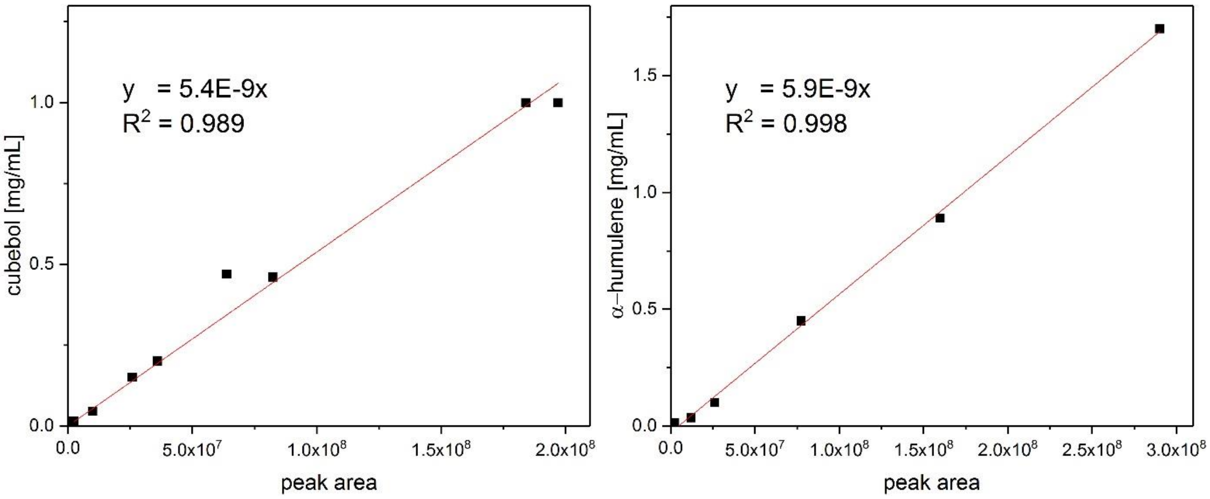

Figure S8: *Calibration curves for GC-FID based sesquiterpene quantification. Different concentrations of cubebol and α-humulene were used for peak-area evaluation via GC-FID.*

References

1. Lin Y-L, Ma L-T, Lee Y-R, Shaw J-F, Wang S-Y, Chu F-H. Differential Gene Expression Network in Terpenoid Synthesis of Antrodia cinnamomea in Mycelia and Fruiting Bodies. J Agric Food Chem. 2017;65:1874–86. doi:10.1021/acs.jafc.6b05386.
2. Engels B, Heinig U, Grothe T, Stadler M, Jennewein S. Cloning and characterization of an Armillaria gallica cDNA encoding protoilludene synthase, which catalyzes the first committed step in the synthesis of antimicrobial melleolides. J Biol Chem. 2011;286:6871–8. doi:10.1074/jbc.M110.165845.
3. Zhou H, Yang Y-L, Zeng J, Zhang L, Ding Z-H, Zeng Y. Identification and Characterization of a δ-Cadinol Synthase Potentially Involved in the Formation of Boreovibrins in Boreostereum vibrans of Basidiomycota. Nat Prod Bioprospect. 2016;6:167–71. doi:10.1007/s13659-016-0096-4.
4. Agger S, Lopez-Gallego F, Schmidt-Dannert C. Diversity of sesquiterpene synthases in the basidiomycete Coprinus cinereus. Mol Microbiol. 2009;72:1181–95. doi:10.1111/j.1365-2958.2009.06717.x.
5. Wawrzyn GT, Quin MB, Choudhary S, López-Gallego F, Schmidt-Dannert C. Draft genome of Omphalotus olearius provides a predictive framework for sesquiterpenoid natural product biosynthesis in Basidiomycota. Chem Biol. 2012;19:772–83. doi:10.1016/j.chembiol.2012.05.012.
6. Yap H-YY, Muria-Gonzalez MJ, Kong B-H, Stubbs KA, Tan C-S, Ng S-T, et al. Heterologous expression of cytotoxic sesquiterpenoids from the medicinal mushroom Lignosus rhinocerotis in yeast. Microb Cell Fact. 2017;16:103. doi:10.1186/s12934-017-0713-x.
7. Quin MB, Flynn CM, Wawrzyn GT, Choudhary S, Schmidt-Dannert C. Mushroom hunting by using bioinformatics: Application of a predictive framework facilitates the selective identification of sesquiterpene synthases in basidiomycota. Chembiochem. 2013;14:2480–91. doi:10.1002/cbic.201300349.
8. Dickschat JS. Bacterial terpene cyclases. Nat Prod Rep. 2016;33:87–110. doi:10.1039/c5np00102a.
9. Rabe P, Schmitz T, Dickschat JS. Mechanistic investigations on six bacterial terpene cyclases. Beilstein J Org Chem. 2016;12:1839–50. doi:10.3762/bjoc.12.173.
10. Baer P, Rabe P, Fischer K, Citron CA, Klapschinski TA, Groll M, Dickschat JS. Induced-fit mechanism in class I terpene cyclases. Angew Chem Int Ed Engl. 2014;53:7652–6. doi:10.1002/anie.201403648.
11. Leeper FJ, Vederas JC. Biosynthesis: Aromatic Polyketides, Isoprenoids, Alkaloids. Berlin, Heidelberg: Springer-Verlag Berlin Heidelberg; Springer e-books; 2000.
